# Sepsis-induced inflammasome impairment facilitates development of secondary *A. baumannii* pneumonia

**DOI:** 10.1080/22221751.2025.2492206

**Published:** 2025-04-09

**Authors:** Aldona Jeznach, Karolina Sidor-Dzitkowska, Magdalena Bandyszewska, Małgorzata Grzanka, Piotr Popławski, Anna Marszalik, Joanna Domagała-Kulawik, Radosław Stachowiak, Grażyna Hoser, Tomasz Skirecki

**Affiliations:** a Department of Translational Immunology and Experimental Intensive Care, Centre of Postgraduate Medical Education, Warsaw, Poland; b Department of Biochemistry and Molecular Biology, Centre of Postgraduate Medical Education, Warsaw, Poland; c Department of Bacterial Physiology, Institute of Microbiology, Faculty of Biology, University of Warsaw, Warsaw, Poland; d Institute of Clinical Sciences, Maria Sklodowska-Curie Medical Academy, Warsaw, Poland

**Keywords:** Sepsis, translational research, nosocomial infection, VAP, inflammasome

## Abstract

Background: *Acinetobacter baumannii* has become one of the most critical pathogens causing nosocomial pneumonia. Existing animal models of *A. baumannii* pneumonia are not relevant to the majority of critical care patients. We aimed to develop a novel model of secondary *A. baumannii* pneumonia in post-sepsis mice. Methods: A two-hit model of sepsis induced by cecal ligation and puncture followed by *A. baumannii* pneumonia on day 5 was established. In addition, the two-hit model was established in humanized mice. A period of 2 h of mechanical ventilation followed by observation was used in additional experiments. Lung histopathology, bacterial cultures, and cellular infiltration were analysed as well as markers of the inflammasome activity *in vivo* and *ex vivo*. Results: *A. baumannii* infection caused mortality and loss of body weight and temperature in post-sepsis mice. Increased lung bacterial burden and dissemination together with signs of enhanced inflammatory injury were observed in post-sepsis mice but not control mice that were challenged with *A. baumannii*. Post-sepsis mice were unable to mount inflammasome activation in response to secondary pneumonia to the level of control mice. Transfer of wild-type but not capsase-1 KO alveolar macrophages was able to restore the pulmonary protection against *A. baumannii*. Mechanical ventilation exacerbated the pathological response to pneumonia in post-sepsis mice but enhanced inflammasome signalling in non-sepsis mice with pneumonia. Conclusions: We established a novel model of *A. baumannii* pneumonia that revealed sepsis-induced impairment of inflammasome activation in alveolar macrophages is critical for the control of secondary *A. baumannii* pneumonia.

## Introduction

*Acinetobacter baumannii* is a ubiquitous, nonfermenting, gram-negative bacterium with a broad repertoire of antibiotic resistance mechanisms [1]. Hospital-acquired pneumonia is the most common clinical form of *A. baumannii infection*, often affecting mechanically ventilated patients in intensive care units (ICUs) [1]. In some ICUs, *A. baumannii* was responsible for up to 26.5% of culture-positive ventilator-associated pneumonia (VAP) in Europe and up to 50% in Asia [2,3].

Sepsis remains one of the major syndromes treated in ICUs, with high mortality exceeding 40% when septic shock occurs [4,5]. Due to the improvement in the early support of multiorgan dysfunctions, most patients survive the early phase of sepsis and succumb to later complications, including nosocomial infections [6]. VAP of *A. baumannii* etiology is a common nosocomial infection in septic patients [7]. Importantly, *A. baumannii* infection is a significant risk factor for mortality among critically ill patients [8]. Despite some progress in the understanding of sepsis-induced immune reprogramming [9], there is a gap in the knowledge about the mechanisms responsible for the development of secondary pneumonia. Human studies characterizing circulating cells indicated that monocytes are generally tolerant in sepsis. Alveolar macrophages (AMs), were shown to have reduced phagocytic and bactericidal properties in murine models of sepsis [10]. Release of TNF and IL-12 upon restimulation of AMs from septic mice was also shown to be reduced [11]. Yet, the resistance of AMs to endotoxin tolerance mediated by GM-CSF and IFN-γ was reported [12]. Alveolar monocytes indeed present higher expression of HLA-DR in comparison to their circulating counterparts, which suggests that the pulmonary milieu can modulate these cells [13].

Mechanisms of host defense to *A. baumannii* are not fully elucidated but involve activation of TLR2, TLR4, TLR9 and NOD1, NOD2 [14–16]. One of the critical mechanisms of innate immunity is inflammasome. The inflammasome is a multiprotein complex that acts as a platform for the activation of caspase-1, of which the active form cleaves pro-IL-1β and pro-IL-18 to generate active cytokines [17]. Active inflammasome also cleaves gasdermin D (GSDMD), the N-terminal fragment of which forms pores triggering pyroptosis. Assembly of the inflammasome is initiated by NF-κB-dependent upregulation of pro-IL-1β and pro-IL18 and NLRs followed by a second signal activating NLR (e.g. K^+^ efflux). There are both structural and functional differences in inflammasome biology between humans and mice; details in our paper [18]. *A. baumannii* was shown to activate the NLRP3 inflammasome in murine macrophages [19]. NLRP3 inflammasome was also reported to mediate lung pathology in a mouse model of *A. baumannii* pneumonia [19], while others found that the NLRP3 inflammasome was protective against infection with a clinical isolate of this bacterium [20]. As most mouse strains are naturally resistant to *A. baumannii* infection, modeling pneumonia of this etiology constitutes a major challenge. Different approaches include the induction of neutropenia, the use of porcine mucin in the inoculum or the use of hypervirulent strains [21]. Each of them has major caveats that limit translational value. The modulation of inflammasome activity in sepsis is complex, and while numerous animal studies have demonstrated its involvement in immunopathology [22–24], clinical observations suggest rather suppression of inflammasome activation during sepsis [25–27].

In this study, we aimed to establish a novel model of secondary pneumonia with *A. baumannii* that resembles the clinical settings in critically ill patients. Furthermore, we wanted to verify whether peritonitis sepsis impairs pulmonary host defense mechanisms, making them susceptible to secondary pneumonia with that opportunistic pathogen. Specifically, we chose to focus on the inflammasome response, which is a rapid and potent mechanism involved in the response to *A. baumannii*.

## Materials and methods

See described methods in the Supplementary file.

## Results

### CLP sepsis predisposes mice to the development of secondary pneumonia with A. baumannii

**Figure 1.**
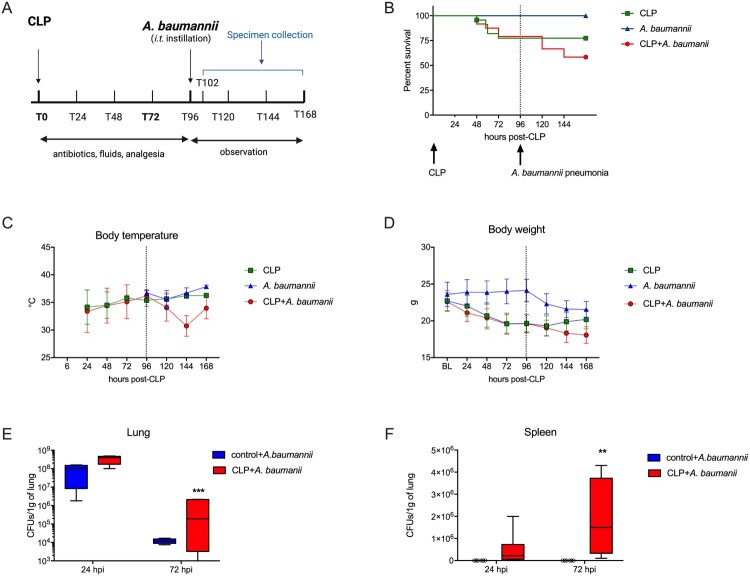
Development of the two-hit model. A. Scheme of the model. B. Survival curves of mice subjected to CLP. B. Changes in body temperature. C. Changes in body weight. E. The results of bacterial cultures from the lung homogenates and F. Spleen homogenates of mice challenged with *A. baumannii*. B-D: n = 20; E, F: n = 6. Data are reported as the means ± SDs. Groups were compared with the Mann–Whitney test. **p < 0.01, ***p < 0.001.

As VAP usually occurs five or more days after ICU admission, we chose to study secondary pneumonia on day five post CLP (Figure 1A). For this, we applied the established CLP model with mortality limited to the first 96 h after surgery [28]. Female mice were used as they are more vulnerable to *A. baumannii* infection [29]. In the study, the *A. baumannii* strain o2251 was used, which exhibited resistance to carbapenems. The resistance mechanism was associated with the presence of *bla_OXA-23_*, *bla_OXA-51_*, and *bla_TEM_* genes (Suppl. Figure 1). Control mice infected with *A. baumannii* did not show any mortality (Figure 1B). However, clinical signs of infection manifested as slight drops in body temperature and weight (Figure 1C, D). In contrast, pulmonary infection with *A. baumannii* in post-CLP mice resulted in late mortality (Figure 1B). Mice with secondary pneumonia presented a sharp decrease in body temperature and persistent loss of weight during the observation period, while the post-CLP mice recovered (Figure 1C, D). Additionally, the two-hit mice had signs of hypocapnia and hypoxemia 24 h after pneumonia. Additionally, in comparison to control mouse pneumonia, they had lower glucose (Suppl. Figure 2). Importantly, infection of post-CLP mice resulted in a higher bacterial burden in the lungs in comparison to infected control mice (Figure 1E). Furthermore, neither of the control mice that received *A. baumannii* had positive cultures from the spleen, while in post-CLP mice challenged with bacteria, there was a dissemination of *A. baumannii* both at 24 and 72 h (Figure 1F). Overall, we established a model of secondary *A. baumannii* pneumonia in post-septic mice that was characterized by impaired clearance of that nosocomial pathogen.

### A. baumannii pneumonia induces lung inflammation in post-septic mice

**Figure 2.**
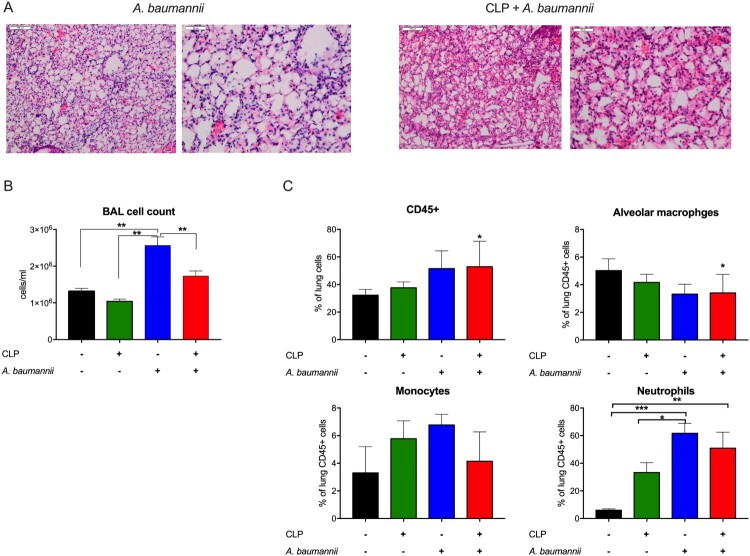
Pneumonia pathology. A. Histopathological assessment *of A. baumannii* pneumonia in control mice (left) and post-sepsis mice (right). B. Total cell count in bronchoalveolar lavage (BAL) fluid. C. Frequencies of the populations of major myeloid cell populations in the lung homogenates of mice. n = 6. Data are reported as the means ± SDs. Groups were compared with ANOVA with Tukey’s post hoc test except for the CD45 + cells and alveolar macrophages figures, where comparisons were made to the healthy control mice (without CLP and without *A. baumannii*) using one-way ANOVA. *p < 0.05; **p < 0.01; ***p < 0.001.

Histopathological examination of lungs from mice infected with *A. baumannii* revealed findings typical of bacterial pneumonia, including diffuse infiltrations, edema and congestions (Figure 2A). In post-CLP mice, secondary pneumonia induced similar changes with a greater magnitude of congestion and fibrin (Figure 2A). However, the total cell count in BAL from the infected post-CLP mice was lower than that in *A. baumannii*-infected mice only (Figure 2B). Flow cytometric analysis showed an increase in the number of leukocytes 6 h after *A. baumannii* instillation in control and post-CLP mice. Interestingly, the percentage of alveolar macrophages decreased in double-hit mice (Figure 2C). Infection with *A. baumannii* induced a strong influx of neutrophils in healthy mice and, to a lesser extent, in post-CLP mice (Figure 2C). The frequency of neutrophils was not significantly higher in the lungs of two-hit model mice than in the lungs of post-CLP mice (Figure 2C). Collectively, these data indicate an impaired inflammatory response in the lungs of post-septic mice to *A. baumannii* challenge.

### Sepsis modulates inflammasome activation in response to secondary pneumonia

**Figure 3.**
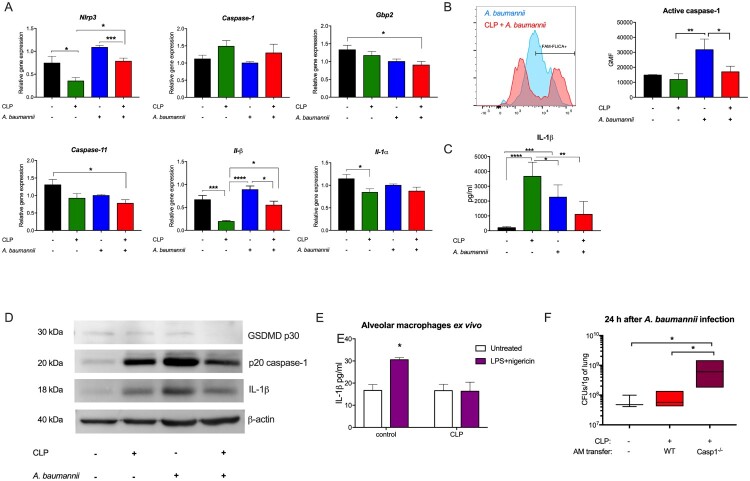
The inflammasome response in secondary pneumonia. A. mRNA levels of the selected inflammasome-related genes were analysed by qPCR and normalized to *Gapdh* expression. B. Activity of caspase-1 in Siglec-F + CD11b^dim^ alveolar macrophages was evaluated with the FAM-FLICA probe. On the left: representative histograms of FAM-FLICA staining in control and post-CLP mice infected with *A. baumannii,* x axis represent intensive of fluorescence and y axis represent number of cells, on right: graph presents the mean values of the geometric mean fluorescence (GMF) for FAM-FLICA. C. Concentration of IL-1β in lung homogenates. D. Immunoblots for the cleaved (active) forms of caspase-1 and gasdermin d (GSDMD) are shown. E. Ability to release IL-1β after *ex vivo* stimulation of alveolar macrophages with LPS and nigericin. F. Transfer of alveolar macrophages from WT, but not Casp1^-/ –^ mice reduced the bacterial load after 24 h in post-septic mice infected with *A. baumannii*. Data are reported as the means ± SDs. Groups were compared with ANOVA with Tukey’s post hoc test. *p < 0.05; **p < 0.01; ***p < 0.001; ****p < 0.0001.

As the differences in recruitment of myeloid cells to infected lungs in healthy vs post-sepsis mice were observed as early as 6 h post challenge, we focused on the inflammasome, which is a rapid response host immunity mechanism. While infection with *A. baumannii* increased the expression of inflammasome-related genes, such as *Nlrp3* (about 1.1) and *Il1*β (about 0.8) in the lungs of previously healthy mice, the transcript levels of these genes were lower in the two-hit mice (about 0.7 for *Nlrp3* gene and about 0.6 for *Il1*β) (Figure 3A). Levels of inflammasome gene expression inform about the transcriptional regulation of this process, but not its activity. Therefore, we assessed the activation of caspase-1 in lung macrophages and found that it was strongly active after infection with *A. baumannii* in healthy but not post-CLP mice (Figure 3B). This was also reflected by the diminished level of IL-1β in the lung homogenates of the two-hit mice in comparison to infected healthy animals (over a threefold decrease) as well as septic mice (over a twofold decrease) (Figure 3C). Moreover, immunoblots confirmed that mice infected with *A. baumannii* activated caspase-1, GSDMD and IL-1 β in their lungs (Figure 3D). However, this response was reduced in post-CLP mice with secondary pneumonia (Figure 3D, Suppl Figure 4, 5). Strikingly, macrophages from post-septic mice were completely incapable of releasing IL-1β upon *ex vivo* stimulation with LPS and nigericin (Figure 3E). Finally, to confirm if inflammasome activity is involved in the control of secondary *A. baumannii* infection, we transferred alveolar macrophages from WT or Casp1^-/ –^ mice to post-CLP mice prior to bacterial challenge. Indeed, transfer of WT, but not Casp1^-/ –^ macrophages, improved bacterial clearance in the lungs of post-septic mice (Figure 3F). The schematic representation of inflammasome activation in sepsis, *A. baumannii* infection, and secondary pneumonia is presented in Suppl. Figure 6 (Suppl. Figure 6). Our findings indicate that miR-223-3p and miR-155-5p play a significant role in controlling NLRP3 expression, highlighting their potential regulatory function in inflammasome activation (Suppl. Figure 7).These results indicate that peritonitis sepsis impairs the capacity to activate the inflammasome in lung macrophages.

### Mechanical ventilation exacerbates secondary A. baumannii pneumonia

**Figure 4.**
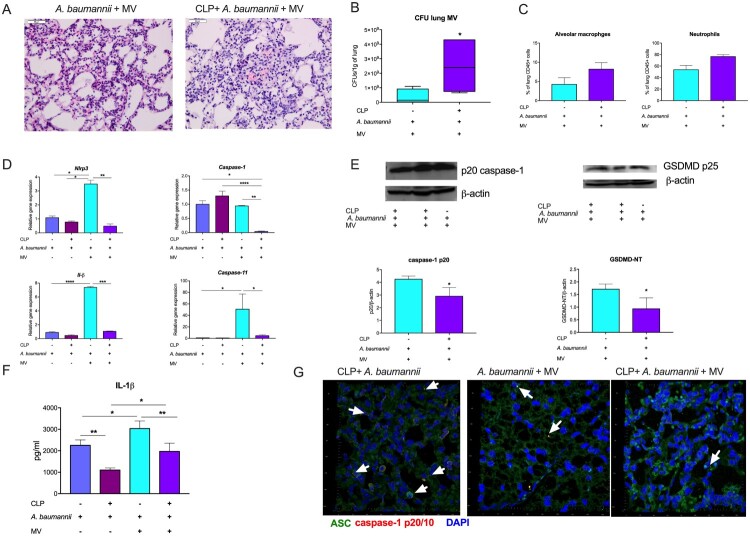
Effects of mechanical ventilation on pneumonia. A. Histopathological assessment of lungs from mice subjected to *A. baumannii* and mechanical ventilation: control (left) and postsepsis (right). B. The results of bacterial cultures from the lung homogenates. C. Frequencies of alveolar macrophages and neutrophils. D. Expression of inflammasome-related genes. E. Immunoblots for the cleaved (active) forms of caspase-1 and gasdermin D (GSDMD). F. Concentration of IL-1β in lung homogenates. G. Immunofluorescence staining for ASC and cleaved caspase-1 in the lungs of infected mice. Arrows indicate puncta of inflammasome assembly (ASC-caspase-1 complex, yellow dots). n = 4-6. Data are reported as the means ± SDs. Groups were compared with Student’s *t* test or ANOVA with Tukey’s post hoc test. *p < 0.05; **p < 0.01; ***p < 0.001; ****p < 0.00001.

Since mechanical ventilation (MV) is known to modulate the pulmonary inflammatory response and, in particular, activate the inflammasome [30], we incorporated 2 h of MV directly after *i.t.* instillation of *A. baumannii*. Then, the mice were allowed to recover, and the lungs were harvested 6 h later. On histological examination, lungs from post-CLP mice that received *A. baumannii* and MV showed more polymorphonuclear infiltrates in comparison to non-septic mice subjected to similar challenge (Figure 4A). Importantly, bacterial clearance in the lungs was impaired in post-CLP mice with pneumonia and MV (Figure 4B). Infection and MV evoked similar changes in the composition of lung leukocytes in both healthy and post-sepsis animals (Figure 4C). MV increased the expression of *Nlrp3* (about 3.4 vs. 1 *without MV*)*, Il1*β (about 7.4 vs. 0.8) and *Casp1* (about 0.9 vs. 1) in infected healthy animals but not in post-septic animals. Mice infected with both CLP and *A. baumannii* exhibited decreased expression of *Nlrp3* and *Casp1* compared to those infected with *A. baumannii* alone (Figure 4D). This was reflected by lower levels of cleaved forms of caspase-1 and GSDMD (Figure 4E). The incorporation of MV increased IL-1β levels in both infected control and post-CLP mice, although it was not reflected in the higher activity of caspase-1 (Figure 4F, G). Additionally, immunofluorescence confirmed the assembly of the inflammasome (ASC-caspase-1 complex) in response to *A. baumannii,* which was modest in the post-CLP mice that received MV (Figure 4G). Although MV modulates the inflammasome response to secondary pneumonia, post-CLP mice consistently display a diminished ability to activate inflammasome.

### A humanized mouse model confirms sepsis-induced impairment of pulmonary immunity

**Figure 5.**
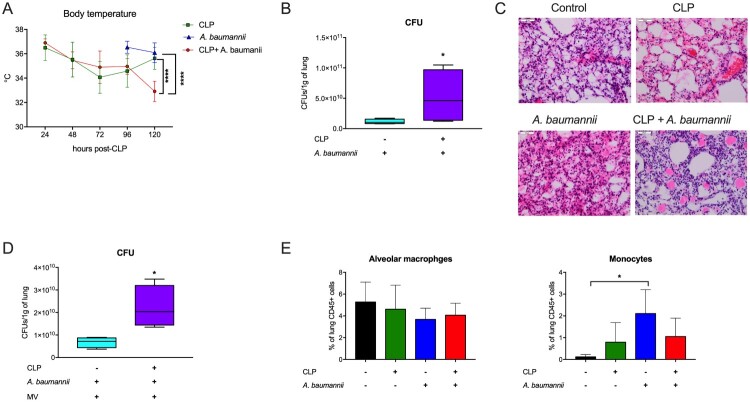
Secondary pneumonia in humanized mice. A. Changes in body temperature, asterisk for comparisons at 120 h. B. The results of bacterial cultures from the lung homogenates. C. Histopathological assessment of lungs from mice subjected to sepsis and/or secondary pneumonia. D. Bacterial cultures from lung homogenates of mice subjected to mechanical ventilation. E. Frequencies of human alveolar macrophages (hCD45 ^+^ hCD206 ^+^ CD169 ^+^ CD14^low^) and monocytes (hCD45 ^+^ CD33^+^ CD14^+^). n = 4-6. Data are reported as the means ± SDs. Groups were compared with the Mann–Whitney test or ANOVA with Tukey’s post hoc test. *p < 0.05; **p < 0.01; ***p < 0.001, ****p < 0.0001.

Humanized mice were generated using a strain with human IL-3 and GM-CSF knock-in, which enhances human myeloid cells differentiation [28]. Our xenotransplantation regimen enabled human chimerism, normal growth of the humanized mice and stable hematological parameters (Suppl. Fig. 3). CLP in these mice induced low mortality (approx. 15%). Instillation of *A. baumannii* induced a decrease in body temperature in both healthy and post-sepsis mice (Figure 5A). Similar to the BALB/c mice, there was a greater bacterial burden in the lungs of post-sepsis humanized mice in comparison to healthy mice challenged with *A. baumannii* (Figure 5B). The lungs of infected post-sepsis (two-hit) humanized mice had severe congestion and greater inflammatory infiltration in comparison to infected healthy humanized mice (Figure 5C). The impairment of bacterial clearance in post-sepsis mice was also evident when MV was applied for 2 h (Figure 5D). Lung infection of humanized mice after 6 h did not change the frequency of human alveolar macrophages (Figure 5E) but increased monocyte infiltration in healthy mice (Figure 5F). Therefore, the humanized mouse model confirms that sepsis impairs pulmonary defense mechanisms against secondary infections and aggravates inflammatory injury.

### Secondary A. baumannii pneumonia is related to an impaired inflammasome response in humanized mice

**Figure 6.**
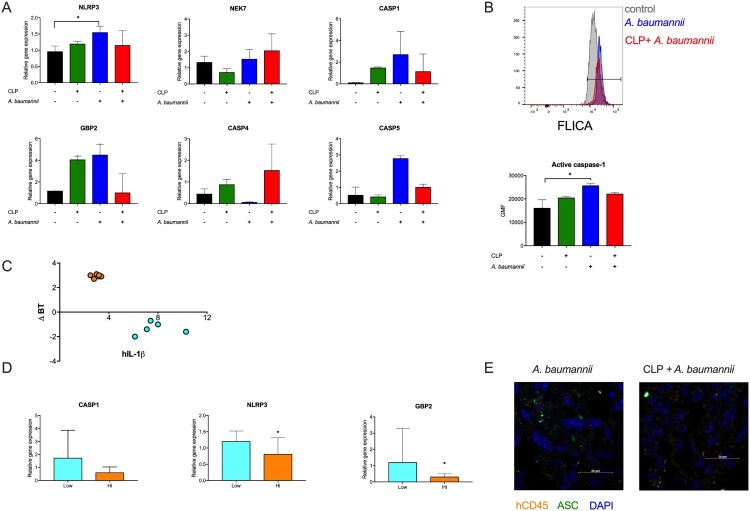
The inflammasome response to pneumonia in humanized mice. A. mRNA levels of the selected inflammasome-related genes were analysed by qPCR and normalized to *Gapdh* expression. B. Activity of caspase-1 in human alveolar macrophages was evaluated with the FAM-FLICA probe. The upper panel shows representative histograms of FAM-FLICA staining in control and post-CLP mice infected with *A. baumannii*, and the lower graph presents the mean values of the geometric mean fluorescence (GMF) for FAM-FLICA. C. Two groups of humanized mice were distinguishable: hypothermic (Low) and normo-/hyperthermic (High) which clustered with the hIL-1β concentration in lung homogenates 6 h after *A. baumannii* instillation. D. Comparison of the expression levels of selected inflammasome genes in regard to body temperature changes. E. Immunofluorescence staining for human CD45 and ASC in the lungs of infected humanized mice. n = 4-6. Data are reported as the means ± SDs. Groups were compared with the Mann–Whitney test or ANOVA with Tukey’s post hoc test. *p < 0.05.

The two-hit model in humanized mice also revealed impaired expression of human inflammasome-related genes in response to a secondary challenge (Figure 6A). The human macrophages in two-hit mice were unable to mount the activity of caspase-1 comparable to pneumonia mice (Figure 6B). Post-sepsis mice 6 h after challenge with *A. baumannii* could be divided based on body temperature change into two groups: low and high temperature (Figure 6C). Interestingly, these groups of mice could also be clustered by the concentration of human IL-1β in their lungs, which was significantly higher in mice that had lower body temperature (Figure 6C). Additionally, the levels of CASP1, NLRP3 and GBP2 transcripts were higher in the lungs of mice exposed to low temperature (Figure 6 D). The formation of the inflammasome in response to *A. baumannii* in humanized mice was also confirmed by immunofluorescence (Figure 6E). These results confirm and expand findings from BALB/c mice that the inflammasome response to *A. baumannii* is impaired in the lungs of post-sepsis mice but also indicate that stronger activation of the inflammasome induces more severe illness.

## Discussion

Here, we successfully established a novel model of secondary *A. baumannii* pneumonia in post-sepsis mice that recapitulates a common clinical scenario. Using a modern clinical isolate resistant to carbapenems enabled the induction of clinically relevant infections, making the model more representative of real patient cases. The resistance mechanism involved *bla_OXA-23_*, *_blaOXA-51_*, and *bla_TEM_* genes, with *bla_OXA-23_* and *bla_OXA-51_* being the most prevalent carbapenem resistance determinants globally, including in Poland [31,32].

Our model was also expanded by the addition of a mechanical ventilation and the use of humanized mice that can recapitulate species-specific responses. The two-hit model enabled the discovery that sepsis impaired the ability to activate the inflammasome in alveolar macrophages in response to *A. baumannii* secondary challenge.

Existing models of *A. baumannii* pneumonia are not relevant to most clinical cases, which occur in nonneutropenic ICU patients. Here, secondary pneumonia was induced after the early sepsis-related death period. Importantly, mortality occurred 96 h after CLP only in mice infected with *A. baumannii*. Secondary infection diminished the clinical recovery of sepsis survivors, which reflects clinical problems in ICU patients [6]. The post-sepsis mice had impaired bacterial clearance in the lungs but were also exclusively susceptible to the dissemination of *A. baumannii*. Two-hit mice also presented more severe lung pathology. Although the two-hit models of peritonitis-induced sepsis and secondary pneumonia were used previously [33,38,39], these models used pathogens with a broader repertoire of virulence factors, such as *P. aeruginosa* or *S. aureus*. Importantly, *A. baumannii* exhibits distinct pathophysiological characteristics in this context, including its ability to evade host immune responses and persist in a post-septic environment [34]. In contrast to *P. aeruginosa* and *S. aureus*, *A. baumannii* induces a weaker inflammasome activation and evades host immune responses through mechanisms like LPS modification and intracellular survival [3,4]. *P. aeruginosa* induces a strong inflammatory response, while *S. aureus*, particularly MRSA, produces virulence factors like PVL, leading to more severe pneumonia [35,36].

The immune response to *A. baumannii* involves activation of the inflammasome, but the role of this mechanism is controversial [19,20]. We found that sepsis impedes the ability to increase the levels of transcripts for inflammasome genes. Importantly, the downregulation of transcripts of the inflammasome genes in the lungs after CLP reflects the findings from the blood of septic patients [26]. Our model revealed that expression levels of most of the inflammasome genes early after the onset of secondary pneumonia is at a similar level to control mice. However, expression of Nlrp3 and IL1β was lower in two-hit mice than *A. baumannii* infected. As the key regulation of the inflammasome activity is at the protein level, analysis of proteins and functional assays were performed. Sepsis reduced the enzymatic activity of caspase-1, its cleavage and the cleavage of IL-1β and gasdermin D. Overall, these results indicate that the mechanism of the inflammasome activity impairment in the two-hit model is regulated at post-transcriptional level and likely involves interactions of different cell types. This stays in line with a report on the altered inflammasome activity regulation in septic patients [27]*.* Our results expand the concept of alveolar macrophages as orchestrators of lung immunity against *A. baumannii* [39]. *Ex vivo* stimulation experiments revealed irresponsiveness of alveolar macrophages from septic mice to classical NLRP3 stimulant, which was chosen to assess specifically the activity of this inflammasome. *A. baumannii* was shown to activate the NLRP3 inflammasome via its outer membrane proteins A and 34 [40,41]. NLRP3 was also required for IL-1β release by *A. baumannii in vitro* [20]. A low level of IL-1β in the lungs of two – hit mice was reflected by a lower number of recruited neutrophils early after pneumonia induction in comparison to non-septic mice, which is likely another mechanism impairing the clearance of pathogens. Importantly, adoptive transfer of only caspase-1^+/+^ alveolar macrophages before secondary infection of post-CLP mice was able to restore the control of bacterial burden in the lungs to the level of healthy mice. Moreover, a protective role of caspase-11 during *A. baumannii* infection was reported [42,43], and the impairment of this mechanism in our model is likely as CLP reduced the expression of caspase-11.

Our model was also reestablished in humanized mice, which were also prone to *A. baumannii* pneumonia following CLP sepsis. Importantly, human macrophages from post-sepsis mice were not able to mount expression of the inflammasome-related genes to a similar level as those from healthy mice in response to bacterial challenge. This was also reflected by the lower activity of capsase-1 in post-sepsis mice than in control mice. Intriguingly, mice that presented hypothermic reactions to secondary pneumonia had higher levels of inflammasome-related genes and higher levels of IL-1β in their lungs. This relationship likely reflects the magnitude of the immune response to invading pathogens that may affect the clinical condition of the organism. Due to low mortality, we were not able to dissect whether this is a protective response in our model. Although humanized mice were previously used to model sepsis [43,44], to our knowledge, this is the first report of a two-hit model in such mice. CLP sepsis was shown to impair hematopoiesis in humanized mice [45], but here, we provide findings about the impairment of human alveolar macrophages. Although the reprogramming of circulating monocytes in human sepsis includes dampening inflammasome activation [25], the existence of such a mechanism in tissue macrophages was speculative.

The expansion of the two-hit model with a period of mechanical ventilation added another layer of translational value to our study. First, *A. baumannii* often colonizes and causes pneumonia in intubated patients. Second, mechanical ventilation alters the pulmonary immune response and perpetuates inflammation [36]. In both BALB/c and humanized mice, mechanical ventilation exacerbated the infection and sepsis-induced inflammasome impairment. In non-septic mice that were infected, mechanical ventilation enhanced inflammasome-related markers, which is in line with the proinflammatory effects of this intervention [36]. Likewise, mechanical ventilation in *S. aureus* and *S. pneumoniae* pneumonia models exacerbated inflammatory lung injury [45,46]. These findings are important for translational research in ICU patients, pointing to the necessity to precisely model different interventions that modulate the immune response. While inhibition of inflammasome activation can be protective against ventilator-induced lung injury, it would likely be harmful in septic patients, as it could further dampen their immunity. However, other common VAP-causing pathogens, such as *P. aeruginosa* and *K. pneumoniae* should be investigated in this context.

Our study has several limitations. First, we were not able to recapitulate the airway dysbiosis and process of colonization that occurs in ICU patients [47]. Second, it should be emphasized that although we proved the important role of the inflammasome impairment in post-sepsis immunoparalysis, other mechanisms such as TLR-signalling downregulation or metabolic reprogramming are likely to simultaneously contribute to the vulnerability to nosocomial infections. We acknowledge that host genetics and comorbidities influence sepsis-induced immune dysregulation, affecting infection susceptibility and immune response variability. Humanized mouse models better reflect human immunity than standard murine models, as they use cells from genetically diverse donors. To capture ICU patient heterogeneity, future studies should include chronic disease models and aged animals, which impact immune function and sepsis outcomes.

## Conclusions

A novel model of secondary *A. baumannii* pneumonia in post-sepsis mice was established. Sepsis impedes pulmonary immunity to this pathogen and specifically impairs the ability of alveolar macrophages to mount a protective inflammasome response. These changes were aggravated by the mechanical ventilation of post-sepsis animals, providing novel immunoregulatory effects of this intervention. The newly developed model can be useful for the dissection of host–pathogen interactions and studying novel therapeutic strategies in a clinically relevant setting.

## Main point summary

Novel two-hit murine model reveals that abdominal sepsis impairs pulmonary immunity by impeding the inflammasome activation to secondary *Acinetobacter baumannii* infection which facilitates development of pneumonia.

## Supplementary Material

Suplement_Jeznach et al 03072025 clean.docx

## Data Availability

The datasets used and/or analysed during the current study are available from the corresponding author upon reasonable request.
