# Supplementary material for: Sepsis-induced inflammasome impairment facilitates development of secondary A. baumannii pneumonia
Source: Emerg Microbes Infect. 2025 Apr 9;14(1):2492206. doi: 10.1080/22221751.2025.2492206 (PMC12016274; doi:10.1080/22221751.2025.2492206)
Supplement: Suplement_Jeznach et al 03072025 clean.docx [file TEMI_A_2492206_SM6182.docx]

**Sepsis-induced inflammasome impairment facilitates development of secondary *A. baumannii* pneumonia.**

Aldona Jeznach^1^, Karolina Sidor-Dzitkowska^1^, Magdalena Bandyszewska^1^, Małgorzata Grzanka^2^, Piotr Popławski^2^, Anna Marszalik^3^, Joanna Domagała-Kulawik^4^, Radosław Stachowiak^3^, Grażyna Hoser^1*^, Tomasz Skirecki(†)^1^

Tomasz Skirecki, head of team and coauthor of this paper, has recently tragically passed away at the age of only 37 years. He was not only a good scientist and anesthesiologist but also our best friend. We dedicate this paper to his memory.

**Supplementary Materials and Results**

**Methods**

*Bacterial inoculum preparation and lung bacterial load assessment*

The clinical *A. baumannii* o2251 strain was isolated from the bronchoaspirate of a patient with VAP hospitalized in the Intensive Care Unit of the Independent Public Clinical Hospital of Prof. W. Orłowski, Centre of Postgraduate Medical Education in Warsaw. This strain exhibits resistance to carbapenems (imipenem and meropenem). The inoculum of *A. baumannii* was prepared by inoculating Luria–Bertani (LB, Sigma) medium with a single colony of bacteria. The culture was grown overnight at 37°C and then brought to the logarithmic phase of growth by dilution to Optical Density OD_600_=0.1 in LB broth medium and growing to OD_600_=0.8 and then concentrated by centrifugation. The bacterial pellet was resuspended to reach a final concentration of 10^9^ CFU/ml. At the indicated time, the mice were sacrificed, and the aseptically excised left lung was weighed, homogenized in PBS and used for determination of CFU/ml following serial dilution on LB agar plates.

*Mechanism of carbapeneme resistance*

The Bacterial & Yeast Genomic DNA Purification Kit (Eurx, Poland) was used to extract genomic DNA from o2251 *A. baumannii* strain following the manufacturer’s protocol. DNA was stored at −20 °C for further study. Amplification of a number of genes encoding common carbapenemases was performed: class D β-lactamases genes *bla_OXA-23_*, *bla_OXA-51_*, *bla_OXA-58_* [1] and *bla_OXA-40_* [2], class A β-lactamases genes *bla_TEM_*, *bla_GES_* [3], class B β-lactamases genes *bla_IMI_*, *bla_IMP_* [4] and *bla_NDM_* [3]. Furthermore, OXA SET A (for *bla_OXA-24_*, *bla_OXA-25_*, *bla_OXA-26_*, *bla_OXA-33_*, *bla_OXA-40_*, *bla_OXA-72_*_)_ and OXA SET C (for *bla_OXA-51_*, *bla_OXA-58_*, *bla_OXA-69_*, *bla_OXA-70_*, *bla_OXA-71_*, *bla_OXA-78_*) primers pairs were used [5]. PCR amplification was performed using the Platinum SuperFi II Polymerase (Invitrogen, MA, USA) in a final volume of 20 µl. PCR was conducted with an initial denaturation step of 2 min at 95°C, followed by 35 cycles of 20 s at 95°C of denaturation, 20 s at 60°C of annealing, 30 s at 72°C of extension and a final extension step of 5 min at 72°C. PCR products were analyzed using gel electrophoresis. Primer data for detected genes are described in Supplementary Table 1.

*Mice*

Mice of the BALB/c strain, RAG-hIL3/GM-CSF (129S4-*Rag2^tm1.1Flv^ Csf2*/*Il3^tm1.1(CSF2,IL3)Flv^ Il2rg^tm1.1Flv^*/J) strain [6] and B6.Cg-*Casp1^em1Vnce^*/J (#032662 [7]) were obtained from The Jackson Laboratories (Bar Harbor, ME, USA). Mice were bred in the animal facility of the Centre of Postgraduate Medical Education (Warsaw, Poland) under pathogen-free conditions with a 12/12 light cycle and fed a standard diet and drinking water ad libitum. All experiments on animals were approved by the Local Ethics Committee no II in Warsaw, Poland and adhered to the ARRIVE guidelines [8]. For microbiological assessment, the mice were sacrificed, and the excised left lung and spleen was homogenized in PBS. For Western blot, qPCR, miRNA and histological analysis lungs were harvested and frozen in -80°C.

*Humanized mice*

Two- to three-week-old female RAG-hIL3/GM-CSF mice were injected i.p. with busulfan (20 mg/kg) (Sigma) for two consecutive days. On the next day, after the second busulfan dose, mice were transplanted with 10^5^ human umbilical cord blood CD34^+^ cells as previously [9]. Eight weeks after transplantation, blood was sampled, and chimerism was assessed by staining with anti-human CD45 (BD) and flow cytometry. Mice with chimerism >5% were considered humanized.

*Two-hit model*

Ten- to twelve-week-old female mice were subjected to peritonitis-derived sepsis by performing cecal ligation and puncture (CLP) surgery [10] as described previously according to the MQTiPSS recommendations [9]. Briefly, all mice received buprenorphine (0.05 mg/kg *i.p*.) 20 min. prior to surgery. Than cecum was ligated at half of its length and punctured twice with a 22G needle and a small amount of cecal content was extruded.. Then, the abdomen was closed with sutures and Histoacryl® tissue adhesive (B. Braun, Aesculap, Germany). From 2 h post-CLP on, mice received subcutaneous therapy: 25 mg/kg imipenem (Fresenius Kabi, Germany) and fluid (1 ml Ringer's solution) with 0.05 mg/kg buprenorphine (Richter Pharma, Austria) every 12 h for five consecutive days. On day five post-CLP, mice were infected with 5 x 10^7^ in 50 μl PBS *A. baumannii* o2251 by oropharyngeal aspiration under isoflurane anesthesia. The mouse was carefully positioned upright using a mouse intubation stand. The tongue was gently lifted with blunt forceps and then moved to one side of the mouth by hand. A pipette was then used to deliver 50 μl of the solution to the back of the tongue. The tongue was held in place until the mouse successfully inhaled the solution. Following the procedure, the mouse was removed from the stand and returned to its home cage, where it recovered within 5 minutes. Mice were evaluated for signs of illness with the modified mouse clinical assessment scoring system (M-CASS) [11]. Rectal temperature was monitored. When the righting reflex was absent and/or the M-CASS score was ≥8, the mice were euthanized.

*Mechanical ventilation*

In addition, experiments, on day five post-CLP, mice were anesthetized with isoflurane and intubated with an 18G catheter (Kent Scientific, CT, USA). Then, the mouse was connected to a mechanical ventilator (SomnoSuite, Kent Scientific) and ventilated in pressure control mode with FiO_2_=0.21, Pinsp=12 cm H_2_O, and PEEP=5 cmH_2_O. After connection to the ventilator, the mice were placed on a heating pad, and body temperature, ECG and oxygen saturation were monitored using a Small Animal Physiological Monitoring System (Harvard Apparatus, MA, USA). Blood pressure was noninvasively monitored using the tail cuff CODA system (Kent Scientific). Each mouse received a bolus of sodium bicarbonicum (0.2 ml) repeated every 30 minutes, and ventilation under isoflurane anesthesia was performed for two hours. Then, anesthesia was stopped, and mechanical ventilation was performed until the mouse was awake. Mice were monitored for another six hours and euthanized.

*Hematoxylin-eosin staining*

Lungs were fixed by intratracheal instillation of 10% paraformaldehyde (PFA, Sigma) buffered in PBS. Then, the lungs were excised and put into 10% PFA for 24 hours and subsequently placed in 70% ethanol. Paraffin-embedded blocks were cut into 4 𝜇m sections. The sections were deparaffinized through three changes of Neo-Clear™ Xylene Substitute (Sigma‒Aldrich) and rehydrated by successive changes of ethanol (absolute alcohol, 96%, 80% and 70%) to distilled water. The slides were stained in hematoxylin (Sigma‒Aldrich) for 10 minutes, rinsed in tap water and stained with eosin (Sigma‒Aldrich) for 5 minutes, washed in water, and dehydrated with four changes of alcohol (70%, 80%, 96% and absolute alcohol, respectively). Slides were cleared with two changes of Neo-Clear™ and mounted with DPX new (Sigma‒Aldrich). The sections were observed by light microscopy (Olympus BX40).

*Immunofluorescence staining*

Slides with four-μm-thick sections of formalin-fixed, paraffin-embedded lungs were kept at 37°C overnight and then deparaffinized by giving three changes in Neo-Clear™ for 10 minutes and two changes for 5 minutes and rehydrated to 50% ethanol by changing an alcohol gradient (100% - 15 minutes, 95% - 5 minutes, 70% - 5 minutes and 50% - 5 minutes). Slides were rinsed with deionized water and placed in PBS buffer for 10 minutes. After rehydration, the slides were subjected to antigen retrieval using proteinase K (A&A Biotechnology, Poland) for 10 minutes at RT, rinsed three times for 5 minutes in PBS and placed in 0.25% buffered Triton X-100. Lung sections were incubated in a blocking solution (PBS containing 2% bovine serum albumin, 1.5% donkey serum, 0.1% Triton X-100) for 30 minutes at RT and incubated with primary antibodies (caspase-1, 1:50, cat # 22915-1-AP, Proteintech; ASC Alexa Fluor® 488 conjugated, 1:100, cat # D2 W8U, Cell Signaling) in blocking buffer overnight at 4°C. Then, the slides were rinsed 3 times in PBS and incubated with secondary antibodies (Alexa Fluor 594 donkey anti-rabbit, cat # A-21207, Invitrogen) for 1 hour at RT. After washing, the tissue slides were incubated with autofluorescence quenching buffer (TrueVIEW® Autofluorescence Quenching Kit, VECTOR Laboratories), and after washing, the slides were counterstained with DAPI (Sigma) and mounted using antifade mounting medium (Vector® TrueVIEW® Autofluorescence Quenching Kit, VECTOR Laboratories). The sections were observed by confocal microscopy (Zeiss LSM800).

*Bronchoalveolar lavage fluid*

After euthanasia, the trachea was cannulated with an 18G vascular catheter (BD), and the lungs were lavaged with 0.5 ml of 2 mM EDTA and 2% BSA in PBS (Sigma) 5 times in total. The BAL fluid was centrifuged at 300 x g for 5 min, and the cells were resuspended in 0.5 ml of PBS. Cells were counted using an automatic cell counter Luna-FX7 (Logos Biosystems, South Korea) and used for flow cytometry staining.

*Cytokine assay*

Mouse and human IL-𝛽 concentrations were analyzed using a commercially available ELISA Kit (Thermo Fisher Scientific, MA, USA) according to the manufacturer’s protocol.

*Hematological and blood gas analysis*

Blood gas analysis was performed using the ABL Flex 90 (Radiometer, Demark). Blood cell counts were analyzed using the BC-2800 Vet analyzer (Mindray, China).

*Ex vivo macrophage stimulation*

Adherent BAL macrophages were seeded in 96-well plates at 4x10^4^ cells per 100 µl. Then, the cells were treated with or without 1 µg/ml LPS (Sigma MO, USA) for 3 h, and 10 µM nigericin (Tocris, UK) was added for the next 30 min. The supernatant was collected and stored at -80°C until the concentration of IL-1β protein was determined by ELISA (Mouse Interleukin-1beta, Invitrogen).

*Alveolar macrophage transfer*

AM were isolated from donors by BAL as described. Then, 2x10^5^ of cells in 50 µl of PBS were given orotracheally to anesthetized C57/BL6 J mice (96 h after CLP). Subsequently, mice were infected with *A. baumannii* and 24 h later lungs were harvested for cultures.

*RT‒PCR and qPCR*

Total RNA was isolated from lung tissue using TRIzol (Ambion, Thermo Fisher Scientific, ~~Massachusetts~~, U.S.). First-strand cDNA was synthesized from total RNA using a RevertAid First Strand cDNA synthesis kit (Thermo Fisher Scientific) for a 100 ng/µl reaction. Real-time quantitative PCR was performed with a LightCycler 480 (Roche, Switzerland) using LightCycler 480 SYBR Green I Master (Roche). Data were normalized to the *Gapdh* mRNA level. Primer sequences are available in Supplementary Table 2.

Total RNA for miRNAs analysis was extracted from lungssamples by miRNeasy Micro Kit (Qiagen, Germany) according to the manufacturer’s recommendations. First-strand cDNA was synthesized from RNA using a miRCURY LNA RT Kit (Qiagen) for a 5 ng/µl reaction. The expression of miR-223-3p (Cat. No.:339306, Qiagen), miR-155-5p (Cat. No.:339306, Qiagen), miR-330-5p (Cat. No.:339306, Qiagen) was quantified by miRCURCY LNA miRNA PCR Assays and PCR Panels (Qiagen) with the use of the specific probes (Qiagen.). Data were normalized to the *5S* mRNA level.

*Flow cytometry*

Analysis of cell phenotype was performed by incubation of 1×10^6^ fresh sample cells with primary antibodies (Supplementary Table 3) at room temperature for 20 minutes. After washing with 2% FBS in PBS, the cells were resuspended in 0.5% paraformaldehyde in PBS. Inflammasome activation was analyzed by incubation of isolated cells with the FAM-FLICA caspase-1 reagent according to the manufacturer’s protocol (ImmunoChemistry Technologies, CA, USA). Cells were analyzed using a FACSCanto II flow cytometer (BD) with Diva software (BD). Analyses were performed with FlowJo software (Treestar, USA). Appropriate negative and FMO controls were used to set gates.

*Immunoblots*

Lung tissues were lysed with T-PER Tissue Protein Extraction Reagent (Thermo Fisher Scientific) buffer. Samples were boiled for 10 min with Laemmli Protein Sample Buffer (Bio-Rad, CA, USA) and separated on 13% SDS‒PAGE gels. Proteins were transferred to PVDF membranes (Bio-Rad), and after blocking with 5% skim milk powder, they were further incubated with the indicated antibodies. For immunoblot analysis, primary antibodies against Caspase1 p20 (Santa Cruz Biotechnology, California, U.S., sc-398715, 1:250 dilution), IL-1 β  (Santa Cruz Biotechnology, sc-515786, 1:200), GSDMD (Thermo Fisher Scientific, PA5-116815, dilution 1:500) and β-actin (Novus, NB600-501SS; 1:1,000 dilution) were used. Secondary antibodies: horseradish peroxidase labeled anti-mouse IgG (H + L, 1:5000, Vector Laboratories, US) for Ab Caspase-1 p20 and Ab β-actin specific, peroxidase labeled anti-rabbit IgG (H + L, 1:5000, Vector Laboratories) for Ab GSDMD. The chemiluminescent reaction was obtained with Clarity Western ECL (Bio-Rad), and pictures were obtained with a UVITEC Mini HD9 (Uvitec, UK). Immunoblotting using a β-actin-specific antibody was used as a loading control. Images have been cropped for presentation; images of uncropped blots are presented in Supplementary results.

*Statistical methods*

The results were analyzed using GraphPad Prism 7 software (La Jolla, CA, USA). The normality of the data was assessed using Shapiro‒Wilk and KS normality tests. The results are presented as the means ± standard deviation (SD) values. Comparisons between groups were performed with a 2-tailed Student’s *t* test or Mann‒Whitney test. Multiple comparisons were tested using one-way ANOVA with Tukey’s post hoc or Kruskal‒Wallis test. Survival curves were compared using the log-rank test. Each experimental group consisted of at least 5 mice, and experiments were repeated at least two times. Graphs present the mean ± SD values. A *P* value below 0.05 was considered significant.

**Supplementary Table 1. Primer sequence for detected genes of carbapenemase.**

| Detected Genes | Primer Sequences (5’-3’) | Product Size (bp) | Reference |
| --- | --- | --- | --- |
| *bla*_OXA-23_ | GATCGGATTGGAGAACCA GA    ATTTCTGACCGCATTTCCAT | 501 | 1 |
| bla_OXA-51_ | TAATGC TTGATCGGCCT TG    TGGATTGCACTTCATCTTGG | 353 | 1 |
| bla_TEM_ | ATAAAATTCTTGAAGACGAAA    GACAGTTAGCAATGCTTAATCA | 1080 | 2 |

**Supplementary Table~~1~~2. Sequences of primes used for qPCR.**

| Species | Name | Sequence 5’-3’ | Source |
| --- | --- | --- | --- |
| HUMAN | GBP2 | F: TTTCACCCTGGAACTGGAAG  R: TGCACAACCGAGGATCATTA | [12] |
|  | CASP5 | F: TTCAACACCACATAACGTGTCC  R: GTCAAGGTTGCTCGTCTCATGG | [13] |
|  | CASP4 | F: GGCAGGACAATGCTTCTTC  R: GACAAAGCTTGAGGGCATCT | This work |
|  | NLRP3 | F: CACCTGTTGTGCAATCTGAAG  R: GCAAGATCCTGACAACATGC | [14] |
|  | CASP1 | F: GGAAACAAAAGTCGGCAGAG  R: ACGCTGTACCCCAGATTTTG | [15] |
|  | IL-1 beta | F: CCAGTGAAATGATGGCTTATTAC  R: CTGTAGTGGTGGTCGGAGATT | [16] |
|  | IL-1 alpha | F: GCATGCCATCACACCTAGTT  R: TTACATATGAGCCTTCCATG | [17] |
|  | GSDMD | F: GTGTGTCAACCTGTCTATC  R: CATGGCATCGTAGAAGTGGAAG | This work |
|  | NEK7 | F: CACCTGTTCCTCAGTTCCAAC  R: CTCCATCCAAGAGACAGGCTG | [18] |
|  | GAPDH | F: ACTTTGGTACGTGGAAGGACT  R: GTAGAGGCAGGGATGATGTTCT | This work |
|  | IL-1β | F: TGAAATGCCACCTTTTGACAG  R: CCACAGCCACAATGAGTGATAC | [19] |
| MOUSE | NLRP3 | F: GGTCCTCTTTACCATGTGCTTC  R: AAGTCATGTGGCTGAAGCTGTA | [20] |
|  | IL-1α | F: GGGAAGATTCTGAAGAAGAG  R: GAGTAACAGGATATTTAGAGTCG | [21] |
|  | GBP2 | F: CTGCACTATGTGACGGAGCTA  R: CGGAATCGTCTACCCCACTC | [22] |
|  | CASP1 | F: AGATGCCCACTGCTGATAGG  R: TTGGCACGATTCTCAGCATA | [23] |
|  | CASP11 | F: TCCAGACATTCTTCAGTGTGGA  R: TCTGGTTCCTCCATTTCCAG | [24] |
|  | GAPDH | F: AGGTCATCCCAGAGCTGAACG  R: CACCCTGTTGCTGTAGCCGTAT | [25] |

**Supplementary Table ~~2~~3. List of antibodies used in flow cytometry**

| Target species | Antigen-fluorochrome | clone | manufacturer |
| --- | --- | --- | --- |
| Mouse | CD45 APC/Fire750 | I3/2.3 | BioLegend |
|  | CD11b AlexaFluor647 | M1/70 | BioLegend |
|  | Gr-1 FITC | RB6-8C5 | BioLegend |
|  | Siglec-F PE | S17007L | BioLegend |
| Human | CD45 BriliantViolet510 | HI-30 | BD |
|  | CD14 FITC | ASR | BD |
|  | CD3 APC-Cy7 | SK7 | BD |
|  | CD20 APC | 2H7 | BD |
|  | CD16 APC-Cy7 | 3G8 | BD |
|  | CD206 PE-Cy7 | 19.2 | BD |
|  | CD169 APC | 7-239 | BD |
|  | CD24 Pacific Blue | ML5 | BD |

~~Results~~

**Supplementary Figure 1. Detection of genes encoding carbapenemases by PCR in o2251 *A. baumannii* strain.** *bla_OXA-23_* (lane 2), *bla_OXA-51_* (lane 3), *bla_TEM_* (lane 4). The molecular size marker (lane 1) is GeneRuler 1 kb DNA ladder (Thermo Fisher Scientific).

**Supplementary Figure 2. Blood gas and critical parameters analysis of mice with pneumonia.** Samples of blood from facial vein were analyzed. A. pH. B. pCO2. C. pO2. D. Glucose concentration. E. Lactates. F. Bilirubin. n=6. Data are reported as means ±10-90 percentile. Groups were compared with Student’s *t*-test. *p<0.05; **p<0.01, ***p<0.001.

**Supplementary Figure 3. Monitoring of mice after xenotransplantation with human CD34+ cord blood cells.** A. Body weight changes (red lines represent the body weight of died mice). B. White blood count. C. Platelet count. D. Red blood count.

**Supplementary Figure 4. Analysis of the activation of the inflammasome related proteins in the lungs of post-sepsis mice.** Densitometric analysis of: A. p20 of caspase-1, B. p30 (N-terminal fragment) of GSDMD, C. p18 of IL-1𝛽. Representative blots are presented in Fig. 3 D. Data are reported as the means ±SDs. Groups (n=5) were compared with ANOVA with Tukey’s post hoc test. *p<0.05; **p<0.01; ***p<0.001.

**Supplementary Figure 5. Western blot analysis of inflammasome proteins in lungs of BALB/c mice subjected to different experimental settings.** Representative Western blots of 1 of at least two or three (with mechanical ventilation) independent experiments are shown.

**Supplementary Figure 6. A schematic representation illustrating the mechanism of inflammasome activation in sepsis (following CLP), *A. baumannii* infection, and secondary pneumonia.** Black arrows indicate changes in gene expression (*italicized font*) and protein activity compared to control mice (without any interventions). Green arrows represent changes in gene expression and protein activity relative to *A. baumannii* infection, while red arrows indicate changes relative to CLP. Created with BioRender.com.


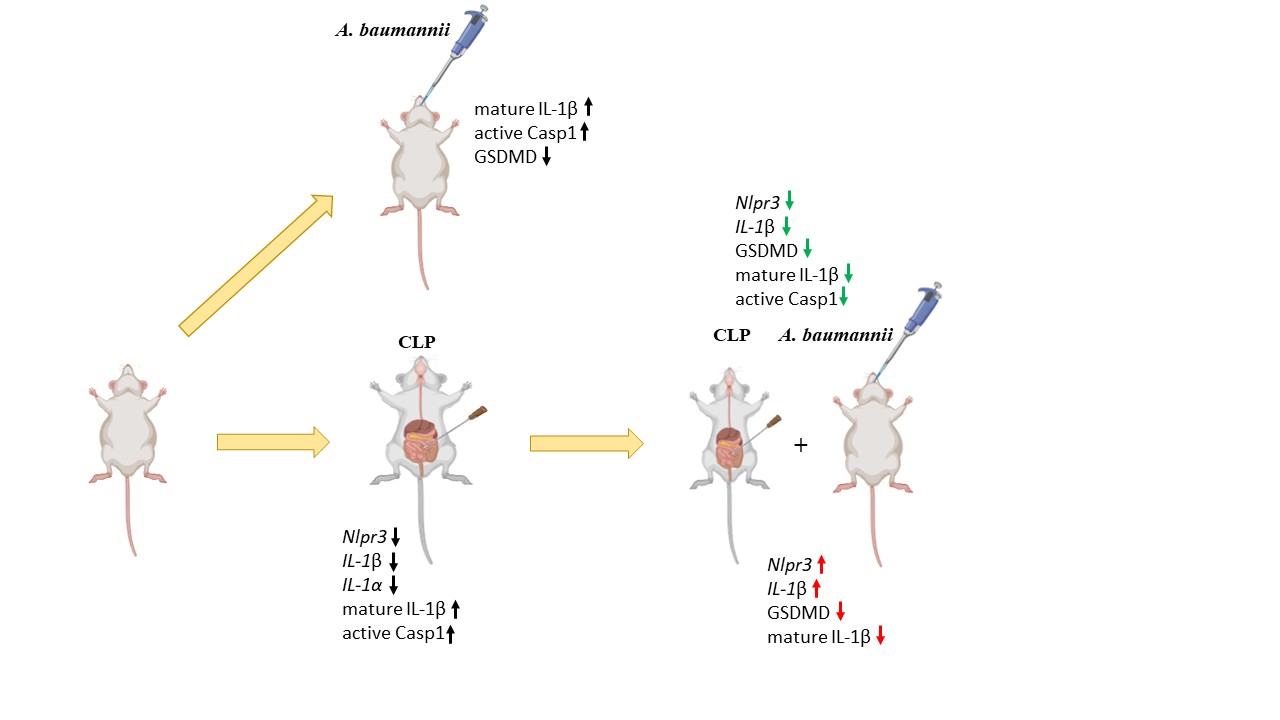


**Supplementary Figure 7. Analysis of miRNAs expression in the lungs of post-sepsis mice.** The expression of miR-155-5p (a), miR-223-3p (b) and miR-330-5p (c) has been measured. Data are reported as the means ±SDs. Groups (n=3) were compared with ANOVA with Tukey’s post hoc test. *p<0.05; **p<0.01; ***p<0.001

**References**

1. Woodford N, Ellington MJ, Coelho JM, Turton JF, Ward ME, Brown S, Amyes SG, Livermore DM. Multiplex PCR for genes encoding prevalent OXA carbapenemases in Acinetobacter spp. Int J Antimicrob Agents. 2006 Apr;27(4):351-3. doi: 10.1016/j.ijantimicag.2006.01.004. Epub 2006 Mar 24. PMID: 16564159.

2. Kasperski T, Romaniszyn D, Jachowicz-Matczak E, Pomorska-Wesołowska M, Wójkowska-Mach J, Chmielarczyk A. Extensive Drug Resistance of Strong Biofilm-Producing Acinetobacter baumannii Strains Isolated from Infections and Colonization Hospitalized Patients in Southern Poland. Pathogens. 2023 Jul 26;12(8):975. doi: 10.3390/pathogens12080975. PMID: 37623935; PMCID: PMC10459043.

3. Han L, Lei J, Xu J, Han S. blaOXA-23-like and blaTEM rather than blaOXA-51-like contributed to a high level of carbapenem resistance in Acinetobacter baumannii strains from a teaching hospital in Xi'an, China. Medicine (Baltimore). 2017 Dec;96(48):e8965. doi: 10.1097/MD.0000000000008965. PMID: 29310399; PMCID: PMC5728800.

4. Serwacki PA, Hareza DA, Kujawska A, Pałka A, Jachowicz-Matczak E, Rybka-Grymek A, Świątek-Kwapniewska W, Pawłowska I, Gniadek Z, Gutkowska K, Gajda M, Wójkowska-Mach J. Molecular epidemiology and clinical significance of carbapenemase genes in carbapenem-resistant Acinetobacter baumannii isolates in southern Poland. Pol Arch Intern Med. 2024 Jun 27;134(6):16734. doi: 10.20452/pamw.16734. Epub 2024 Apr 23. PMID: 38656082; PMCID: PMC11615936.

5. Adams-Haduch JM, Paterson DL, Sidjabat HE, Pasculle AW, Potoski BA, Muto CA, Harrison LH, Doi Y. Genetic basis of multidrug resistance in Acinetobacter baumannii clinical isolates at a tertiary medical center in Pennsylvania. Antimicrob Agents Chemother. 2008 Nov;52(11):3837-43. doi: 10.1128/AAC.00570-08. Epub 2008 Aug 25. PMID: 18725452; PMCID: PMC2573138.

6.Willinger T, Rongvaux A, Takizawa H, et al. Human IL-3/GM-CSF knock-in mice support human alveolar macrophage development and human immune responses in the lung. Proc Natl Acad Sci U S A 2011; 108:2390-5.

7.Rauch I, Deets KA, Ji DX, et al. NAIP-NLRC4 Inflammasomes Coordinate Intestinal Epithelial Cell Expulsion with Eicosanoid and IL-18 Release via Activation of Caspase-1 and -8. Immunity 2017; 46:649-59.

8. Kilkenny C, Browne WJ, Cuthill IC, Emerson M, Altman DG. Improving bioscience research reporting: the ARRIVE guidelines for reporting animal research. PLoS Biol 2010; 8:e1000412.

9. Skirecki T, Drechsler S, Hoser G, et al. The Fluctuations of Leukocytes and Circulating Cytokines in Septic Humanized Mice Vary With Outcome. Front Immunol **2019**; 10:1427.

10. Wichterman KA, Baue AE, Chaudry IH. Sepsis and septic shock--a review of laboratory models and a proposal. J Surg Res 1980; 29:189-201.

11. Shrum B, Anantha RV, Xu SX, et al. A robust scoring system to evaluate sepsis severity in an animal model. BMC Res Notes **2014**; 7:233.

12. Makovski V, Jacob-Hirsch J, Gefen-Dor C, et al. Analysis of gene expression array in TSC2-deficient AML cells reveals IRF7 as a pivotal factor in the Rheb/mTOR pathway. Cell Death Dis **2014**; 5:e1557.

13. Zhang J, Brodsky IE, Shin S. Yersinia Type III-Secreted Effectors Evade the Caspase-4 Inflammasome in Human Cells. bioRxiv **2023**.

14. Oliviero F, Zamudio-Cuevas Y, Belluzzi E, et al. Polydatin and Resveratrol Inhibit the Inflammatory Process Induced by Urate and Pyrophosphate Crystals in THP-1 Cells. Foods **2019**; 8.

15. Yang Q, Liu Q, Lv H, Wang F, Liu R, Zeng N. Effect of pulegone on the NLPR3 inflammasome during inflammatory activation of THP-1 cells. Exp Ther Med **2020**; 19:1304-12.

16. Mambwe B, Neo K, Javanmard Khameneh H, et al. Tyrosine Dephosphorylation of ASC Modulates the Activation of the NLRP3 and AIM2 Inflammasomes. Front Immunol **2019**; 10:1556.

17. Cervin Serrano S, Gonzalez Villareal D, Aguilar-Medina M, et al. Genetic polymorphisms of interleukin-1 alpha and the vitamin d receptor in mexican mestizo patients with intervertebral disc degeneration. Int J Genomics **2014**; 2014:302568.

18. Wolf S, Wu W, Jones C, Perwitasari O, Mahalingam S, Tripp RA. MicroRNA Regulation of Human Genes Essential for Influenza A (H7N9) Replication. PLoS One **2016**; 11:e0155104.

19. Lai JL, Liu YH, Peng YC, et al. Indirubin Treatment of Lipopolysaccharide-Induced Mastitis in a Mouse Model and Activity in Mouse Mammary Epithelial Cells. Mediators Inflamm **2017**; 2017:3082805.

20. Zhao W, Shi CS, Harrison K, et al. AKT Regulates NLRP3 Inflammasome Activation by Phosphorylating NLRP3 Serine 5. J Immunol **2020**; 205:2255-64.

21. Altmeier S, Toska A, Sparber F, Teijeira A, Halin C, LeibundGut-Landmann S. IL-1 Coordinates the Neutrophil Response to C. albicans in the Oral Mucosa. PLoS Pathog **2016**; 12:e1005882.

22. Finethy R, Luoma S, Orench-Rivera N, et al. Inflammasome Activation by Bacterial Outer Membrane Vesicles Requires Guanylate Binding Proteins. mBio **2017**; 8.

23. de Castro-Jorge LA, de Carvalho RVH, Klein TM, et al. The NLRP3 inflammasome is involved with the pathogenesis of Mayaro virus. PLoS Pathog **2019**; 15:e1007934.

24. Zeis T, Allaman I, Gentner M, et al. Metabolic gene expression changes in astrocytes in Multiple Sclerosis cerebral cortex are indicative of immune-mediated signaling. Brain Behav Immun **2015**; 48:313-25.

25. Chen F, Barman S, Yu Y, et al. Caveolin-1 is a negative regulator of NADPH oxidase-derived reactive oxygen species. Free Radic Biol Med **2014**; 73:201-13.
